# Beyond Storytime: Oklahoma Public Libraries’ Comprehensive Approach to the Resilience of Refugee Children and Their Families Support

**DOI:** 10.3390/ijerph22081298

**Published:** 2025-08-19

**Authors:** Salma Akter, Suchismita Bhattacharjee

**Affiliations:** Division of Interior Design, University of Oklahoma, Norman, OK 73019, USA; s.surma@ou.edu

**Keywords:** public libraries, refugee children, resilience, community integration, Oklahoma, inclusive design

## Abstract

Public libraries serve as vital community hubs that foster engagement, empowerment, and education, particularly for vulnerable populations, including refugee children and families. This study examines how Oklahoma’s public libraries contribute to refugee resilience and identifies challenges they face in providing these essential services. Using a qualitative method approach, including 20 semi-structured interviews with library staff, questionnaire surveys, and observations conducted across three Oklahoma library systems (Metropolitan, Pioneer, and Tulsa City-County) the study explored programs, services, and strategies that support refugee adaptation and integration. Findings reveal that libraries excel in three key areas: cognitive services (language literacy, digital access, educational resources), socio-cultural services (community building, cultural exchange), and physiological services (safe spaces, welcoming environments). These services contribute to building human, social, and economic capital, with human capital consistently ranked as most crucial for refugee resilience. However, libraries face significant challenges, with language barriers, program gaps, and outreach limitations being the most prevalent obstacles. Additional barriers include facility constraints, transportation difficulties, resource limitations, and privacy concerns. The study proposes nine comprehensive guidelines for creating sustainable pathways to refugee resilience through enhanced library services, emphasizing proactive community engagement, staff training, multilingual resources, advocacy, strategic partnerships, tailored programming, transportation solutions, cultural competence, and welcoming environments. This study contributes to understanding how public libraries can function as inclusive institutions that support refugee children’s successful integration and development in their new communities.

## 1. Introduction

Public libraries serve as dynamic community hubs, fostering engagement, empowerment, and education for individuals of diverse backgrounds [1]. They prioritize education through various means, leveraging both human expertise and technological advancements. Beyond building social capital, public libraries have proven instrumental during pre- and post-migration phases, serving as a vital information source for displaced and refugee communities [2].

Refugees navigating resettlement encounter numerous challenges, from language and cultural barriers to employment skills gaps. Despite potential discrimination in host countries [3], public libraries offer crucial opportunities for refugees to acquire new languages and skills, promoting integration and empowerment [4,5]. Libraries organize multicultural activities like storytelling sessions for refugee families [6], provide acculturation and citizenship services [2] and some even hire social workers to address refugees’ basic needs [7,8]. Recent literature portrays public libraries as democratic spaces [9] that support refugees [10] by addressing accessibility, financial constraints and staff training needs [11,12,13]. Johnston et al. [14] found that public libraries in Hungary and Poland responded to an influx of Ukrainian refugees by addressing community needs, rooted in their core values of inclusivity and community engagement. Libraries also contribute to the UN Sustainable Development Goals, especially in advancing education (SDG 4) and reducing inequalities (SDG 10), through culturally responsive programs for refugee families [15]. For instance, in Lisbon, Portugal, library patrons initiated multicultural storytelling sessions among refugee families to support cultural acclimation [6]. In Scotland, tailored information support has been adequate for Syrian refugees at different stages of resettlement, stated [16] in their study. Some libraries have further expanded support by hiring social workers to assist refugee patrons with complex needs, including housing and trauma recovery, have stated in Lloyd [7] and Garner et al.’s [17] studies.

While numerous studies highlight libraries’ transformative role in fostering refugee acculturation and integration [18], research gaps remain. Existing studies often focus on discrete aspects of library services without comprehensive examination of how libraries contribute to refugee resilience and well-being. Some researchers have highlighted libraries’ role in achieving Sustainable Development Goals (SDGs) [19,20] but offer no specific design model for implementation. Lloyd [21] emphasized health literacy programs for refugees but noted insufficient empirical evidence on refugees’ perceptions of library services. Salzano et al. [22] explored integrating refugees’ cultures into library design but maintained a narrow focus.

Additionally, few studies integrate perspectives from both library staff and refugee patrons, constraining the development of practical, inclusive design frameworks. For instance, while Lloyd [21] discusses health literacy and information resilience, few empirical models exist to guide libraries in holistically supporting refugee well-being [23]. Grace and Sen [23] and Varheim [24] acknowledge the resilience-building potential of libraries through services like employment, literacy, and small business support, but their focus is often on natural disasters or generalized populations rather than refugee experiences. Together, these gaps highlight the need for research that connects refugee-specific needs with the ways libraries can foster long-term, adaptive resilience. Additionally, there is a lack of practical, actionable guidance for designing inclusive learning environments that help refugees navigate vulnerability, displacement-related stress, and insecurity in host countries [25]. Furthermore, there are critical gaps in the existing literature, including the limited integration of refugee-centered perspectives, lack of practical design frameworks, and minimal attention to the physical environments of libraries, which play a significant role in inclusion and well-being. DeJonge [26] emphasizes the role of spatial settings in refugee integration. These gaps underscore the need for research that holistically examines how public libraries can support refugee resilience. Addressing this gap requires a nuanced understanding of how libraries’ services, stakeholder experiences, and spatial features interact to shape inclusive environments during resettlement.

In the United States, public libraries provide vital services to refugees and asylum seekers through children’s programs, literacy initiatives, and digital learning opportunities [11]. Libraries can monitor local immigration demographics [27], offer culturally sensitive services, support English language development, and build connections with local organizations [28,29]. Despite these efforts, significant gaps persist in meeting refugees’ information needs. Research on Hispanic/Latino [30] and Chinese [31] communities provide some insights, but exploration of other refugee groups remains limited. Challenges include insufficient multilingual resources, inadequate staff training, and difficulties with outreach.

The current literature inadequately explores how public libraries holistically support refugees with acculturation and language acquisition, often highlighting only fragments of information needed by refugees [32,33,34]. While previous research examines psychological integration in learning environments to address refugees’ anxiety and trauma [35], few studies explore how public libraries specifically contribute to refugee resilience.

This issue is especially urgent in Oklahoma, a key refugee resettlement state that has received increasing numbers of displaced families from Myanmar, Afghanistan, Syria, and the Democratic Republic of Congo [36]. While public libraries in Oklahoma have piloted programs such as ESL tutoring, citizenship classes, and children’s literacy events [37], many operate with limited funding, staffing, and institutional support—challenges that disproportionately affect rural branches. Yet few studies have examined how libraries in low-resource settings like Oklahoma navigate these barriers to serve refugee communities effectively.

Building on the identified gaps in the literature, this study investigates how public libraries can serve as inclusive, resilience-building environments for refugee communities during resettlement. Focusing on the case of Oklahoma, it examines the roles of library services, stakeholder experiences, and spatial design in fostering integration and well-being. The study specifically explores how public libraries support refugee resilience, the challenges they encounter, and the strategies they employ to create sustainable, trauma-informed, and culturally responsive services. The research is guided by the following questions:

RQ 1. What specific programs and services do public libraries in Oklahoma offer to support refugee communities?

RQ 2. What challenges do public libraries in Oklahoma face in providing services to refugee communities?

RQ 3. What are the most effective services provided by public libraries in Oklahoma, and what success stories illustrate their impact?

RQ 4. What comprehensive guidelines and strategies can be implemented within public libraries to create sustainable pathways for refugee resilience?

Using a qualitative case study approach comprising multiple methods, combining interviews, surveys, and observations across Oklahoma’s library systems, this study aims to identify effective strategies for enhancing refugee resilience. With a comprehensive literature review, the research illuminates the evolving role of public libraries in refugee resettlement.

The subsequent sections delve into theoretical foundations and methodology, followed by findings organized according to the research questions. The discussion provides insights into proposed service and design models, concluding with a summary and reflective analysis.

## 2. Theoretical Underpinnings

This study draws on three interrelated theoretical frameworks, refugee vulnerability, integration, and community resilience, to examine how public libraries foster refugee well-being during resettlement. Refugees often encounter systemic barriers in host societies, including restricted access to information, language challenges, discrimination, and unfamiliar institutional landscapes [38,39]. These obstacles can hinder adaptation and recovery, underscoring the need for accessible, trusted, and community-based support systems such as public libraries.

### 2.1. Refugees’ Vulnerability in a New Country

Refugees navigating their new environments face a complex array of challenges, including unfamiliar systems, institutions, and cultural norms. They often experience prejudice from both society and government policies. Research indicates that factors such as personal attributes, including religious and cultural beliefs, literacy, and social support, contribute to their resilience [40]. Yu et al. [41] noted that refugees have lower employment rates and incomes. Lamba [42] observed that their job quality for refugees in Canada is often poorer than in their home countries.

However, barriers such as language difficulties, racism, and discrimination can significantly undermine this resilience [38]. Access to reliable information is crucial for maintaining resilience during and after displacement [39]. A lack of information about education, health, housing, and employment can hinder refugees’ ability to integrate into their new communities.

### 2.2. Public Library Supports Refugee Integration and Multidimensional Needs in a New Country

Public libraries support refugees’ information needs, search behaviors, and adaptation to new cultures to successfully integrate into their host countries [16,43]. The past literature suggests that immigrant integration is a two-way process, with adaptation necessary by both settlers and host societies where public libraries can play an inevitable role [44,45,46,47].

Shepherd, Petrillo and Wilson [45] highlighted that a public library can contribute to the three-stage integration process of newcomers in a new country: transitioning (dealing with immediate issues), settling in (becoming participative members of society), and finally reaching a stage where newcomers understand where to find information. Public libraries can mitigate information access bottlenecks that change over time.

Public libraries play a crucial role in fostering human growth, enhancing identity, and empowering refugees [6]. They serve as educational spaces, promoting literacy and facilitating social gatherings [48]. They provide a safe environment for refugee adolescents [26] to meet strangers and build bridging social ties [49]. As universalistic public spaces, they offer opportunities for informal social contact [10,50] and support immigrants’ political integration through discussion and debate [10].

Libraries help newcomers feel part of the community, fostering trust between users and staff and increasing information-seeking behavior [10]. They provide language literacy programs, job training, political debates [51], and summer enrichment programs [52]. Additionally, libraries offer other activities like initial preparation to foster a sense of community and welcome refugees [11,53,54].

While personal qualities like religious beliefs and social support contribute to refugee resilience [40], trusted information sources, including public libraries, play a crucial role during and post-displacement [39]. However, the role of public libraries in promoting community resilience, including that of refugees, remains largely unexplored [23].

### 2.3. Conceptualizing Refugee Resilience Through the Public Library Lens

Resilience reflects a system’s capacity to endure and adapt to disturbances and changes, encompassing its ability to absorb shocks, recover, and transform [55,56,57]. This concept involves mechanisms for managing adverse conditions and adapting to new circumstances [58,59]. It encompasses not only recovery but also the ability to evolve in response to social, political, and environmental changes [60,61]. Central to resilience is adaptive capacity, the ability to leverage human, economic, social, and environmental resources to respond to disruption [62]. This capacity is shaped by broader spatial, political, and institutional conditions [63,64,65].

Community resilience expands this concept to collective systems, defined by [66] as “the existence, development, and engagement of community resources by community members to thrive in an environment characterized by change, uncertainty, unpredictability, and surprise.” It emphasizes shared values, networks, and resources that enable collective adaptation. Key contributors include social capital, governance structures, and inclusive infrastructure [58,67]. Aldrich & Meyer [68] emphasize social capital as essential for post-crisis recovery. However, the role of public libraries within this framework remains underexplored [23]. Veil and Bishop [69], building on Norris et al. [59], identify four key community capacities—economic development, information and communication, social capital, and community competence—as pillars of resilience, with public libraries aligning closely with each.

Among the many definitions of community resilience, study adopts Norris et al.’s [59] framework to conceptualize refugee resilience as a networked process, shaped by institutional and infrastructural supports. For refugees—who often face social exclusion, economic hardship, and limited access to services—public libraries can foster resilience by offering inclusive, resource-rich environments that enhance both individual capacity and collective belonging. It also aligns with Berkes and Ross [58], who emphasize shared values, governance, and infrastructure as foundations of resilience. For refugee populations—often facing housing instability, economic insecurity, and social isolation—libraries can serve as key enablers of resilience by fostering social cohesion and offering inclusive spaces for learning and collective action.

Informational resilience is foundational for refugee adaptation in a new country. It helps individuals navigate unfamiliar systems, overcome language barriers, and access critical services such as healthcare, legal aid, housing, and employment [16]. Public libraries serve as trusted, non-judgmental spaces that enhance information literacy [70], health literacy [19] and facilitate incidental knowledge sharing found in public libraries in Queens, New York [71]. These spaces support refugees’ communicative integration by enabling access to local media, language learning, and culturally relevant resources [72]. Informational resilience also extends to everyday practices, such as tutoring, child play, and intergenerational reading, which foster a sense of familiarity and inclusion in the host society [73,74].

Social resilience is equally vital. Libraries act as inclusive civic spaces that promote bonding and bridging social capital through language classes, cultural events, and educational programming [10]. These initiatives mitigate isolation, foster cross-cultural relationships, and support broader processes of social integration found in Diaz′s [72] research. Studies in both North America and Europe find that libraries enhance social participation and civic trust, especially for immigrant women and families [40,75].

Economic and political integration is facilitated through skill-building workshops, digital literacy classes, and access to civic information [12].These programs not only improve employability but also promote political awareness and engagement, key indicators of long-term inclusion and resilience [72].

Finally, personal integration is advanced through the therapeutic functions of libraries as calm, welcoming environments that support emotional well-being. Their design and programming promote belonging and agency, aligning with trauma-informed principles of safety and dignity [69,76].

Together, these domains reveal the multifaceted ways public libraries contribute to refugee resilience. By drawing on community resilience theory and integration models, this study positions libraries as holistic infrastructures of care, where refugees not only access resources but also reconstruct their identities, rebuild social ties, and foster a sense of home in new environments.

### 2.4. U.S. Refugee Resettlement Ceilings and Public Libraries’ Role in Refugee Integration

Since the establishment of the U.S. refugee resettlement program under the Refugee Act of 1980, the annual ceiling on refugee admissions has been set by the President in consultation with Congress. This ceiling determines the maximum number of refugees who may be admitted to the United States each fiscal year. For fiscal year 2025, the ceiling was set at 125,000, consistent with the three prior years. Refugees are defined as individuals unable or unwilling to return to their country of origin due to persecution or a well-founded fear of persecution based on race, religion, nationality, membership in a particular social group, or political opinion [77].

Historical data reveals fluctuations in both refugee ceilings and admissions. Notably, fiscal years 2013 to 2016 saw admissions closely align with the ceiling, around 70,000 to 85,000 refugees annually. The lowest recorded ceiling was 15,000 in FY 2021, which was subsequently raised to 62,500. Since 1975, the United States has welcomed over 3 million refugees, with approximately half originating from Asia [78].

Public libraries serve as essential community anchors [79] in refugee resettlement and integration efforts. Libraries provide welcoming, inclusive spaces where refugees can access information, resources, and programs tailored to their unique needs. The American Library Association [80] underscores the importance of equitable access, affirming through its Bill of Rights that library services must be open to all regardless of origin, age, or background [80].

Despite their pivotal role, libraries face ongoing challenges related to consistent funding and resource allocation, which can lead to regional disparities in service availability. Sustained support is necessary to ensure that public libraries continue to function effectively as vital hubs of integration, empowerment, and social connection for refugees and immigrant communities [81].

## 3. Design, Methodology, and Approach

This study employed a qualitative methods approach, integrating semi-structured interviews, questionnaire surveys, and observation techniques. The research was situated in Oklahoma and Tulsa counties, selected due to their high concentration of refugees over the past decade. The Pioneer, Tulsa, and Metropolitan library systems were chosen for this investigation.

### 3.1. Contextual Understanding and Rationality

Oklahoma has emerged as a key destination for refugees in the United States, particularly in Oklahoma City and Tulsa. Between 2003 and 2015, Oklahoma City saw 1495 refugees resettled, while Tulsa welcomed 1152 individuals, with significant numbers also settling in Jenks, Guymon, and Broken Arrow. Burmese refugees have constituted the predominant majority, comprising 80% of Oklahoma’s refugee population during this period.

The state’s public libraries—most notably the Metropolitan Library System, the Pioneer Library System, and the Tulsa City–County Library (TCCL)—play a vital role in supporting these refugee communities. They provide essential resources such as multilingual collections, access to information on local services, and specialized programs. For refugees, access to computers and the internet at these libraries is especially critical for maintaining connections with family abroad, pursuing educational opportunities, and navigating the job market.

The Tulsa City–County Library (TCCL) is the largest system in the state, with 24 branches, a collection of more than 1.7 million items, and services that include Wi-Fi, a bookmobile, homebound delivery, and specialized collections. It has been nationally recognized as a “5-Star Library.” The Metropolitan Library System, based in Oklahoma County, is the second largest, comprising 19 libraries that serve residents, students, and property owners. The Pioneer Library System (PLS) operates 12 branches across Cleveland, Pottawatomie, and McClain counties and maintains a reciprocal borrowing arrangement with the Metropolitan Library System. Although smaller in scale, PLS has demonstrated a strong commitment to inclusivity by providing programs and resources that specifically support refugees and immigrants [82].

While Oklahoma’s public libraries are geographically widespread, their distribution reveals a rural–urban divide. Many smaller communities, particularly those with fewer than 10,000 residents, rely on unaffiliated libraries with limited services, while metropolitan areas such as Oklahoma City and Tulsa are served by large, resource-rich systems (Oklahoma Department of Libraries, 2022) [82]. This uneven landscape underscores the critical role of the Tulsa City–County, Metropolitan, and Pioneer Library Systems in providing consistent, inclusive, and comprehensive support to refugee and immigrant populations concentrated in Oklahoma’s urban centers [82]. 

These three library systems were selected for this study because of their size, geographical coverage, and demonstrated commitment to inclusivity and community engagement, which make them particularly relevant for understanding how public libraries support refugee resilience in Oklahoma.

### 3.2. Participant Recruitment

Purposeful and snowball sampling methods were employed to recruit interviewees. The researchers considered 20 library staff members as key informants. The process involved telephone outreach and email communication. Before participant recruitment, a pilot study was conducted on libraries to identify specific libraries in Oklahoma.

The library systems were selected using the Oklahoma Department of Libraries website, and a desk study was conducted to identify key locations with significant refugee populations. Tulsa and Oklahoma County were determined as the primary research areas. The researchers shortlisted 20 libraries based on refugee concentration and created a database of librarians’ profiles with contact information.

For telephone outreach, the researchers contacted the front desk of each library system, explained the project’s aims, and sought experts working in refugee services. For email correspondence, they detailed the project’s aims, objectives, and IRB approval number, requesting additional potential interviewees through snowball sampling. They then sent interview questions, schedules, and consent forms. The final step involved sending a Zoom link or physical location details for the interview.

**Figure 1:**
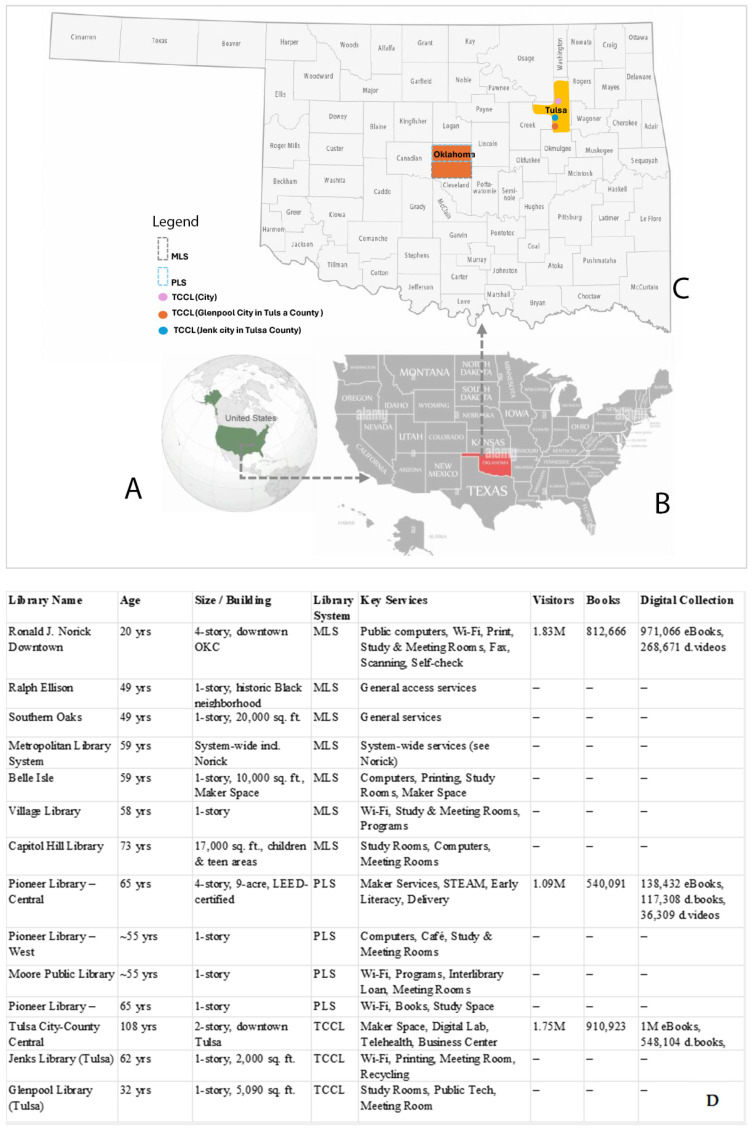
(**A**) US in World map; (**B**) Oklahoma in US map; (**C**) Oklahoma County and Tulsa County in Oklahoma State; (**D**) Library Snapshot. Source: The author interpreted all maps and snapshots based on data from the Oklahoma Department of Libraries Statistics (oklahoma.gov) [82].

This meticulous approach ensured the recruitment of informed and willing participants, thereby enhancing the depth and reliability of the qualitative data collected. Twenty participants from twenty libraries under three library systems were recruited. All participants were aged between 30 and 50, with four males and sixteen females. Their designations ranged from the children’s development section manager to the marketing department/outreach section. All had more than five years of work experience in libraries. Table 1 and Figure 1 show the participant’s profiles and the Libraries’ snapshots.

### 3.3. Data Collection

This study collected data between July and October 2023 through semi-structured interviews, site observations, and a follow-up questionnaire. Twenty interviews were conducted—four in person and sixteen via Zoom—each lasting 47 to 50 min and conducted in English. The study was approved by the Institutional Review Board (IRB) at the University of Oklahoma.

Following the interviews, an online questionnaire gathered descriptive data on library characteristics (e.g., size, building age, staffing). Site visits complemented these data through observation, photography, and detailed fieldnotes. The interviews were guided by 15 questions organized under five key themes: (1) lifelong learning and personal development, (2) language acquisition and literacy, (3) information and communication during emergencies, (4) economic capital, and (5) social support and community connection. These themes addressed the following overarching question: How do public libraries support refugee children’s learning, development, and integration?

Participants discussed how libraries promote personal growth and language acquisition, offer critical information during emergencies, and support refugee families’ legal and economic needs. Libraries also emerged as sites of social connection, offering networking opportunities, emotional support, and community-building activities. To assess long-term impact, participants reflected on questions such as the following: “What effect do libraries have on the human capital of young refugees?, “Have you observed sustained benefits or success stories?”. Participants also relayed feedback from refugee families on the libraries’ contributions to their human, social, and economic development—offering insight into the broader and lasting influence of library services on refugee communities.

### 3.4. Data Analysis

The data analysis was conducted through hand and tool-based methods. Recorded transcripts underwent a rigorous cleaning process, with two authors reviewing and correcting them. The cleaned data was sent to each interviewee for clarity verification as part of member checking.

Thematic analysis involved systematically identifying, analyzing, and reporting patterns within the collected data. This process included open, axial, and selective coding to explore common issues affecting refugees’ adaptiveness to social, cultural, and economic resilience in resettlement. During axial coding, connections between categories were explored to understand how these themes relate to one another and the broader research questions. This phase explored the why and how of category relationships and their impact on understanding refugees’ resilience. This involved examining causal relationships, contextual factors, and variations within the data. The selective coding phase synthesized the findings into overarching themes that directly aligned with the study’s research aims, focusing on the library’s role in refugee adaptability and resilience-building. Codes, categories, and themes were generated through comprehensive text analysis using both manual systems and NVivo for graphical visualization.

While the lead author conducted the primary analysis, all co-authors collaboratively developed and finalized the search terms and coding protocols. Thematic coding was directly guided by the research questions and was refined through multiple revisions to improve coherence, minimize redundancy, and uphold analytic consistency.

### 3.5. Trustworthiness and Validity

To enhance validity and credibility, the data analysis underwent multiple revisions to reduce overlap. Data quality was ensured through constant comparative analysis (how they are comparable) and negative case analysis (what is left) [83]. Peer debriefing [84] further enhanced the credibility of the findings. The lead author invited one peer with relevant experience, specifically in refugee-related work and inclusive public library design, to review the codebook and emerging themes. This external validation ensured the interpretations were logical, consistent, and aligned with the study’s research goals. Peer debriefing, member checking, and author’s flexibility were conducted to ensure internal data validity.

Peer debriefing was conducted with one academic peers who specialize in refugee integration and library science; they reviewed a sample of coded transcripts and thematic summaries to question assumptions, identify potential biases, and offer critical feedback. Member checking involved sending synthesized thematic narratives and selected quotations to the participants for confirmation. The feedback was used to refine language and verify the authenticity of interpretations. The author also practiced methodological flexibility by iteratively adapting the interview protocol based on early interview insights—for example, expanding probes around health care support and youth agency after participants emphasized these areas. This responsive approach ensured that emerging concerns from participants were meaningfully integrated into both the data collection and analysis process.

## 4. Findings

The findings are organized according to the study’s four research questions, each addressing a different dimension of how public libraries in Oklahoma engage with refugee communities. These include (1) the specific programs and services offered; (2) the challenges libraries face in implementation; (3) the effectiveness of services and notable success stories; and (4) proposed guidelines and strategies to foster long-term refugee resilience through library infrastructure and programming.

### 4.1. RQ1—What Specific Programs and Services Do Public Libraries in Oklahoma Offer to Support Refugee Communities?

The findings indicate that public libraries in Oklahoma function as inclusive, multidimensional community anchors that support refugee integration through a spectrum of targeted programs and services. Seven key themes emerged from participant interviews: (1) Cognitive Inclusion and Lifelong Learning, (2) Economic Empowerment and Career Readiness, (3) Socio-Cultural Belonging, (4) Political and Civic Integration, (5) Health and Well-being Services, (6) Physiological Safety and Space Access, and (7) Community Partnerships and Outreach.

#### 4.1.1. Cognitive Inclusion and Lifelong Learning

We found that many libraries actively promote educational inclusion for refugee children and families by offering ESL classes, early literacy programs, and bilingual story times. Children benefit from regular access to homework help, STEM activities, and engaging reading programs designed to foster curiosity and school readiness.

*“We’re one of the few places where you don’t need a card or ID to get online. That’s a lifeline for many.”*

(PLS 15)

Several librarians emphasized the role of digital access—such as free Wi-Fi, public computers, and mobile hotspots—as foundational to educational participation, particularly for refugees who may lack home internet or devices. Even when formal programming was limited due to staff or space constraints, libraries worked to maintain resource corners or informal learning environments.

##### Digital Access and Technology Integration

All library systems prioritize digital access as a cornerstone of community service. As one librarian noted, “We’re one of the few places where you don’t need a card or ID to get online. That’s a lifeline for many” (PLS 15). This emphasis on digital access was echoed in every library system. Free public Wi-Fi, computer access, and, in some locations, Wi-Fi hotspot lending programs, ensure that refugee families—especially those lacking home internet—can engage in critical tasks such as job applications, schoolwork, and communication with extended family. Some libraries also integrate maker spaces and technology-rich environments to stimulate hands-on creativity and digital literacy, although space constraints limit these offerings in smaller branches. Libraries embrace innovation through maker spaces, which serve as dynamic hubs for creativity and hands-on learning. One librarian shared,

“Lately, our maker spaces are accessible for all ages and allow for kids to get started, as well as teens and adults” (PLS 5). Unfortunately, maker spaces are not included in some libraries due to space limitations.

##### Educational Resources and Programs

We found that Oklahoma’s public libraries offer diverse educational resources that support refugee learning and integration. These include STEM programs, language learning tools like Mango, financial literacy workshops, digital skills training, tutoring, homework help, and career preparation services. Participants described these resources as tools for personal growth and self-improvement. One librarian noted, “We do offer all kinds of resources in ways that you can improve yourself” (PLS 20). Others highlighted targeted academic support: “We offer resources for exam preparation and tutoring services, helping individuals succeed” (PLS 2). STEM activities—such as robotics and 3D printing—were especially engaging for refugee youth, fostering curiosity and future-oriented thinking. As one staff member shared, “Kids are excited about robotics and coding—it gives them a chance to explore and think about future careers” (PLS 9).

##### Language Learning and Literacy Support Initiatives

Language and literacy support further extend this mission. Many libraries are expanding their world language collections to include Vietnamese, Farsi, Dari, Pashto, and Zomi, reflecting the linguistic needs of diverse refugee communities- as noted by PLS 3, “Our catalogers are going through and developing world language collections”. English learning remains a cornerstone, with libraries partnering with literacy organizations to offer ESL classes for all age groups. As one librarian shared, “We partner with the Community Literacy Center to offer English as a Second Language classes at various branches” (PLS 15). Another Librarian shared “We have public space for people who are teaching classes for any age to use whenever they schedule it” (PLS 12).

Programs target both children and adults in refugee communities. “There is a program for the smallest children, which is playtime and story time, so they can be exposed regularly to other kids and stories” (PLS 12). For adults, libraries provide resources to improve language skills and gain employment skills. Bilingual programs, such as story times, make resources more accessible to non-English speaking families. Libraries promote early literacy through specialized programs like bilingual story times. PLS 16 stated, “Our playtime and story time sessions introduce young children to language and literacy in a fun, interactive setting, laying the foundation for future learning”.

#### 4.1.2. Economic Enhancement and Career Development

Libraries provide job resources, skill-building programs, and career development opportunities. Many libraries offer resources for resume building and job applications, including databases like Job Now and Learning Express Library. Personalized assistance through services like “Schedule a Librarian” provides one-on-one help with resume writing and job applications (PLS 14).

Workforce development is supported by partnerships with organizations such as Goodwill Industries, providing comprehensive job readiness training. Entrepreneurship and small business support are prioritized with resources and workshops on business plan development, funding opportunities, and mentorship programs.

Libraries host job fairs and networking events and provide tailored training sessions in digital literacy and Microsoft Office skills (PLS 2). They also offer skill-building workshops on financial literacy, coding, and technology. Libraries provide volunteer and internship opportunities that build professional skills, particularly for teens and young adults.

For refugees, libraries offer specialized support such as multilingual assistance and integration programs for children. “Bilingual volunteers help refugees with job applications and summer programs integrate their children into the community” (PLS 14).

Some libraries provide housing navigators to help community members with housing applications and connect individuals with local services for food, shelter, and medical care.

“In some of our libraries, in the downtown library, a couple of days a week, we have a housing navigator to help members of our community”

(PLS 15).

#### 4.1.3. Socio-Cultural Services and Facilities

Public libraries play a significant role in providing socio-cultural services that foster inclusion, cultural expression, community interaction, and learning opportunities across age groups. These services are particularly valuable for marginalized and resettled populations, including refugees, who often lack access to culturally sensitive and socially enriching environments.

##### Inclusivity and Welcoming Atmosphere

We found that libraries actively foster an inclusive environment where individuals from all backgrounds feel respected and empowered. As one participant noted, “Three of the values of our library are to welcome, empower, and respect. And this applies to all people in our communities” (PLS 3).

##### Programs for All Ages

We found that libraries offer age-inclusive programming, intentionally creating space for different developmental and social needs. As a participant shared, “We have toddler reading, teen spaces, children’s spaces” (PLS 1), highlighting how programming fosters a love of learning across the lifespan.

##### Facilitating Social Connections

Libraries also served as spaces for building social connections among families. Children’s programs were seen as key opportunities for community building: “Our children’s programs are a really good way for them to socialize with different families in the area” (Interview_PLS 10). Another participant explained, “We try to be a community hub… where people do feel” (Interview_PLS 14), emphasizing the relational role of the library.

##### Cultural and Linguistic Inclusivity

Participants described various efforts to support cultural diversity and multilingual engagement. Public events—such as “traditional Mexican dances” during Hispanic Heritage Month (Interview_PLS 16)—were seen as opportunities to celebrate identity and create intergroup understanding. Libraries also introduced programs to “*learn about different religions and other diverse populations*” (Interview_PLS 9), underscoring their role as platforms for intercultural education.

#### 4.1.4. Political and Civic Engagement

Public libraries act as civic anchors, equipping immigrant communities with the knowledge and tools to navigate complex bureaucratic and political systems. Participants described how libraries not only host formal citizenship classes but also curate culturally responsive materials that foster civic learning. As one librarian explained, “Our Citizenship Corner received a grant last year… to provide materials for people looking to obtain their citizenship” (PLS 6), underscoring how targeted resources support immigrants’ long-term settlement goals. Another librarian noted assisting patrons with nuanced, real-life concerns, such as “how to register to vote or renew passports” (PLS 12), demonstrating libraries’ practical role in enabling civic participation. For many immigrant families, the library becomes a trusted space to access critical, often inaccessible, information: “I’ve seen families come in and use our citizenship display and study guides” (PLS 6). These findings illustrate how libraries contribute to democratic inclusion and empower newcomers to engage with the broader sociopolitical landscape.

#### 4.1.5. Health and Well-Being Services

Libraries serve as non-stigmatizing entry points for health education and emotional support, especially for populations with limited access to formal healthcare systems. As one librarian shared, “We offer mental health classes at our library here at Central” (PLS 19), highlighting the institution’s role in mental health literacy. Programs that blend health and social connectedness—such as family yoga, meditation sessions, and reading circles—were described as avenues for building routine, community, and resilience. “We have a meditation series and various fitness classes, such as yoga and Taichi…,” explained another participant (PLS 8), emphasizing how libraries meet holistic well-being needs. Programs like “Circle of Parents” provide culturally sensitive parenting support, addressing the psychosocial needs of both children and caregivers (PLS 4). These narratives underscore the therapeutic and preventive health functions of public libraries, particularly within displaced and underserved communities.

#### 4.1.6. Physiological Inclusion Through Facilities

Libraries serve as vital “third places” that prioritize dignity, safety, and belonging for diverse communities, including refugee families. Librarians consistently described their branches as physically and emotionally secure environments where families feel welcome and children can freely engage in unstructured activities. One participant noted, “We’re a big draw for a gathering place where they know their kids are safe to do what they want” (PLS 18), highlighting the importance of low-pressure spaces especially for trauma-impacted youth.

For Zomi refugee families, specific branches such as Jenks and Glenpool act as trusted sanctuaries. Libraries also actively reduce social and financial barriers to access; as one librarian stated, “We’re one of the few places… you can go to without the expectation of spending money” (PLS 17). Some libraries offer after-school programs and “teen hangout time” (PLS 18), while others provide “cool zones” during extreme weather (PLS 9) and outdoor spaces where children can “run around and play games” (PLS 19).

Participants emphasized that libraries are among the safest public places for community gathering without financial burden. As one librarian put it, “We’re one of the few places in the community that you can go to without the expectation of spending money” (PLS 17). Another added, libraries create “a great, safe, enjoyable space for [people] to have fun and participate in all of the variety of programming that we offer” (PLS 11).

#### 4.1.7. Community Partnership and Outreach

Libraries strategically collaborate with local organizations to extend their reach and deepen their cultural responsiveness. Participants highlighted partnerships with literacy centers, faith-based organizations, and refugee support agencies to deliver English classes and wraparound services. “We partner with the community literacy center to offer English as a second language classes at various branches” (PLS 15), one staff member explained. Others emphasized cross-sector collaboration with groups like Spero, Care, and Catholic Charities: “We have key community groups doing that very specific work in our community” (PLS 11), suggesting that libraries serve as both conveners and facilitators. Engagement also extends beyond library walls, with librarians participating in school events, festivals, and USCIS support centers to ensure broader access: “We were there with an information table [at the USCIS Afghan Support Center]…” (PLS 17). These findings demonstrate how libraries function as relational infrastructures—creating pathways to belonging, visibility, and trust across multiple systems.

### 4.2. RQ 2—What Challenges Do Public Libraries in Oklahoma Face in Providing Services to Refugee Communities?

**Figure 2:**
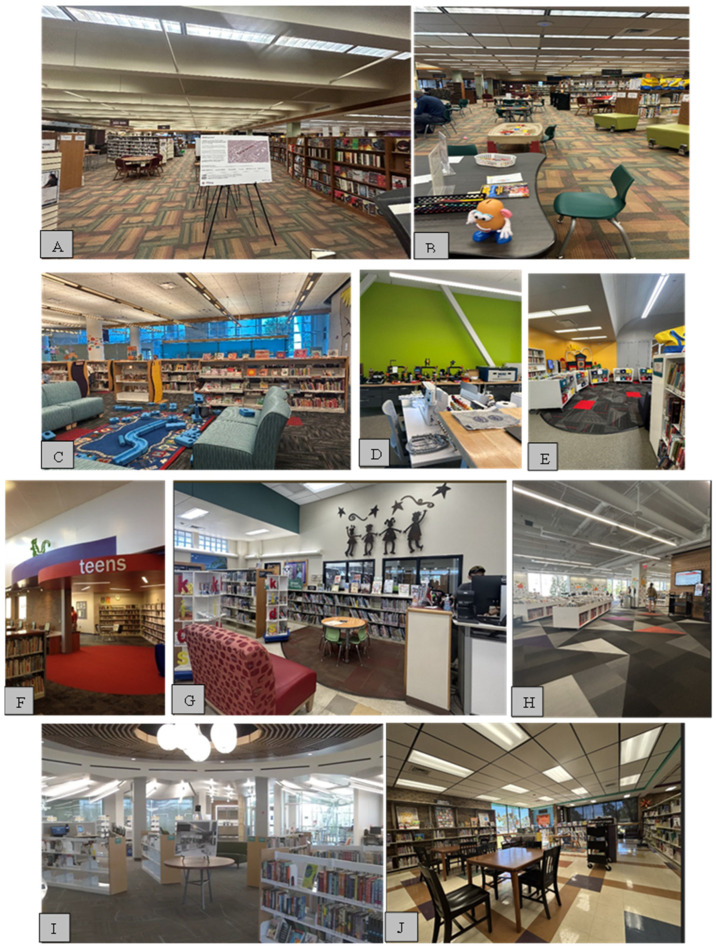
Space and facility constraints: (**A**,**B**) Moore Public Library; (**C**) Downtown Library; (**D**) Pioneer Library-Central; (**E**) Pioneer Library-West; (**F**) Southern Oak Library; (**G**) Glenpool Library Tulsa; (**H**,**I**) Tulsa Central Library; (**J**) Jenks Library, Tulsa. (Source: Author Field work, 2023).

Data analysis identified seven key challenges that public libraries in Oklahoma face when providing services to refugee communities. The list of major challenges indicated by the library staff during the interview is noted in Table 2 and Figure 2.

#### 4.2.1. Facility Limitation and Space Constraints

We found that physical infrastructure often limits libraries’ ability to serve refugee patrons effectively. Many smaller branches operate within buildings that are decades old, with inadequate space, poor signage, and insufficient technological infrastructure. One librarian explained, “Our building is about 55 years old (Figure 2A,B). Finding enough spaces that can accommodate people and their technology, such as their laptops, is difficult” (PLS 6). Others noted that study rooms, quiet areas, and child-friendly spaces are lacking or poorly delineated. Signage is another critical issue. Many libraries have poor or inadequate signage, making it difficult for patrons to navigate the facilities. Available signage is often only in English, presenting a barrier for non-English-speaking patrons, as mentioned by one of the library staff.

Furniture placement and overall spatial design were also concerns. In some branches, furnishings were described as outdated or inappropriately arranged, diminishing the comfort and usability of spaces (Figure 2A–C,G). In contrast, more modern branches such as Pioneer Library West were seen as examples of functional, inviting design, offering ergonomic furniture, vibrant aesthetics, and even amenities like a coffee shop (Figure 2E). In Tulsa, the City Center Library also ensures clear furniture placement with aesthetic appropriateness to attract the patrons.

#### 4.2.2. Transportation Challenges

Transportation significantly impacts refugees’ accessibility to public libraries. In Oklahoma City and Tulsa, public transportation systems are notably inadequate. The poor state of public transit makes commuting difficult, particularly for families who do not live within walking distance of libraries. The transit system in Tulsa is described as weak and disconnected, with limited stops that fail to cover the entire city efficiently. These transportation issues extend to other areas like Moore, where transportation system improvements have yet to meet community needs, and Norman, which lacks intercity public transportation altogether. As one of our respondents eloquently stated, “The bus routes sometimes don’t make sense… transportation can be an issue” (PLS 10). Spanish-speaking families and other communities often rely on a single car, which becomes an obstacle when multiple family members need to travel simultaneously. The location of libraries plays a crucial role; those in residential areas or within walking distance provide easier accessibility. However, many libraries are not ideally placed, limiting access for communities that rely on public transport.

#### 4.2.3. Resource Constraints: Funding and Staffing

Financial limitations were repeatedly identified as a major obstacle. As one librarian stated, “Funding is always an obstacle” (PLS 11). Limited budgets constrain the ability to offer multilingual resources, hire specialized staff, and run culturally responsive programs. The high cost of translation services and the need to prioritize basic operational expenses, such as salaries and materials, further compound the issue. Staffing shortages and turnover create instability, limiting program continuity and innovation. Several participants emphasized the pressure of doing more with less. “Time and staffing are the biggest obstacles” (PLS 19), one staff member remarked, pointing to the systemic strain placed on human capital. There is a clear desire to develop specialized programs for skills development, yet barriers in staffing and expertise present challenges. “I don’t know how to get to how to learn a specific skill for a job class” (PLS 2) reflects the need for enhanced resources and staffing to create and implement these programs effectively.

#### 4.2.4. Limited Awareness and Outreach

A critical challenge lies in the visibility and understanding of library services within refugee communities. Many refugees are unfamiliar with what libraries offer beyond book lending. “The number one obstacle is awareness” (PLS 2), one librarian explained, while another added, “Providing information or distribution of the information to everyone is a great challenge” (PLS 4). Misunderstandings about the library’s role—such as confusion with bookstores—further complicate outreach. Trust is also a factor, particularly when refugees feel misunderstood or undervalued. “Some don’t consider people who are peripheral to the larger community” (PLS 16), one participant noted, reflecting how exclusionary perceptions can limit engagement. Gaps in multilingual resources can hinder service effectiveness, as emphasized by the statement, “there are some barriers there for sure that exist” (PLS 19).

#### 4.2.5. Cultural and Contextual Understanding

We found that a lack of cultural responsiveness significantly impacts refugees’ sense of belonging. Many libraries lack staff who share linguistic or cultural backgrounds with the communities they serve. This gap limits culturally grounded engagement and impedes relationship-building. One staff member candidly noted, “We hope that we can find a way to connect with the community of refugees” (PLS 5). Additionally, the absence of programming tailored to refugee experiences—such as trauma-informed services or culturally relevant materials—was seen as a missed opportunity. As one librarian shared, “Offering in-depth training or programs may be a challenge for some libraries” (PLS 8). Libraries frequently lack programs specifically tailored to refugee needs, as noted, “we have not done programming at my location… that is specifically geared toward refugee children or their families” (PLS 9).

#### 4.2.6. Language and Cultural Barriers

Language emerged as one of the most persistent barriers. Limited multilingual materials restrict access, as noted by a member of the Downtown OKC library, “We’ve struggled to find material in Pashto and Dari” (PLS 1). This scarcity hampers libraries’ ability to support multilingual needs effectively. The absence of bilingual staff compounds this issue, which was indicated during the interview, “language is one of the biggest barriers” (PLS 16). Another concern highlighted was the limited availability of English language learning resources. “We don’t specifically have English language learning classes at all our libraries” (PLS 3). This gap underscores the challenges faced in addressing language barriers, which are further exacerbated by the lack of bilingual staff in many branches.

#### 4.2.7. Gaps in Programming and Specialized Services

Public libraries face challenges in supporting refugee and immigrant communities due to limited specialized training and programming. For example, a librarian from the Downtown Library noted, “We don’t do any training on starting businesses” (PLS 1). Similarly, the Moore Library lacks training related to refugee trauma, with a staff member stating, “We don’t have any training on refugee trauma and how to communicate with someone who’s been through that” (PLS 7). While some interviewees mentioned “refugee trauma,” it is important not to assume all refugees have experienced trauma or require trauma-specific support. Providing trauma recovery is beyond library staff roles; however, there is a clear need for trauma-informed communication and cultural sensitivity training to improve services. One participant noted such training “may be a challenge for some libraries” due to limited resources (PLS 8), which can lead to missed opportunities to better support these communities. Additionally, the Northeast Side of Oklahoma City Library reported no programming specifically for refugee children or families: “We have not done programming specifically geared toward refugee children or their families” (PLS 9). Vocational training gaps were also common, with multiple staff confirming they “do not formally teach vocational training” (PLS 12, 18). Several libraries acknowledged the general absence of skill-building programs tailored to refugees.

#### 4.2.8. Data and Privacy Concerns

Libraries encounter significant challenges due to the lack of data and awareness about refugee needs. The Belle Isle Library struggles with tracking refugee usage and feedback, as indicated by the fact that “we don’t track what people are searching for or reading” (PLS 12). There is a general lack of demographic and usage data which was also noted by another library staff member, “we don’t have a lot of demographic data tied to specific services” (PLS 2). This absence of information impedes libraries’ ability to tailor services effectively. “The number one obstacle is awareness, like communicating to the refugee communities what resources we have available” (PLS 2).

**Figure 3:**
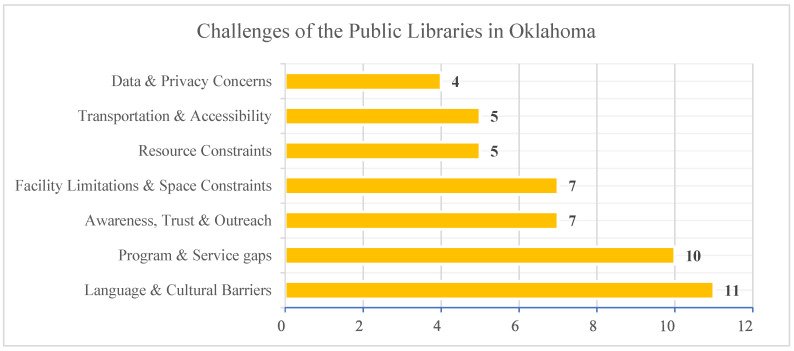
Challenges identified by the public library for refugee integration.

Overall, we found that public libraries in Oklahoma face a range of challenges in supporting refugee and immigrant communities (Figure 3). Among these, language and cultural barriers were the most frequently cited issues, noted by 11 respondents. This was followed by program and service gaps (10 responses), suggesting a need for more inclusive and targeted offerings. Facility and space constraints and issues related to awareness, trust, and outreach were each cited by seven respondents, highlighting both physical limitations and challenges in community engagement. Fewer libraries reported concerns related to resource constraints, transportation and accessibility, and data and privacy concerns. These findings emphasize the complex; multilayered nature of barriers faced by public libraries and point to the need for systemic improvements in both infrastructure and culturally responsive service delivery.

### 4.3. RQ 3—What Are the Most Effective Services Provided by Public Libraries in Oklahoma, and What Success Stories Illustrate Their Impact?

Across interviews, a consistent hierarchy emerged: participants overwhelmingly identified human capital as the most critical area of library support, followed by social capital and then economic capital. These categories were not perceived in isolation but as mutually reinforcing, with many participants emphasizing the interdependencies among them.

#### 4.3.1. Human Capital: Skill-Building and Learning as Foundational Support

Human capital was consistently ranked as the most vital form of support provided by libraries. Eight interviewees placed it first, pointing to services that develop individual capacity, language proficiency, and civic readiness. English as a Second Language (ESL) classes, citizenship preparation, adult literacy programs, and digital literacy workshops were frequently cited as transformative. One librarian explained, “I will start with the human capital because I think that’s important for them to be accustomed to, be more comfortable in, it before going to a job. Because they go into a job without being comfortable. It makes it harder, challenging, and more challenging for them in the long run. Training during that time and making them feel welcome can be done all at the same time” (PLS 13). Another librarian reflected, “I think judging by the types of programs and services that I saw offered at my library system, I would say the human capital because all the things like the citizenship classes, the introductory English as a Second Language classes, and a lot of the job help and the literacy programs, all of that I think points back to that” (PLS 16).

The emphasis on human capital was echoed in the direct ranking statements of multiple interviewees: “Humans are first, then social second, and economics is third” (PLS 3); “Human first, social second, and economic third” (PLS 1); “Humans are almost tied at the top, and economics might be the third” (PLS 11); and “Human capital, you mentioned gaining knowledge” (PLS 14). Furthermore, one participant underscored the importance of human capital specifically for younger refugees: “Human capital will be first for young refugees” (PLS 9).

These services were seen not only as tools for learning but also as mechanisms for healing, rebuilding self-efficacy, and establishing trust in a new society. Human capital development, particularly among youth and newly arrived families, was considered essential to long-term integration and resilience.

#### 4.3.2. Social Capital: Building Relationships and Community Belonging

Social capital was generally viewed as the second most important domain, although some respondents ranked it even higher. Participants described how libraries fostered informal networks, interpersonal trust, and a sense of community belonging. Key services included conversation circles, cultural programming, children’s story times, and other events that encouraged interaction across cultural and linguistic boundaries. Several interviewees stressed the importance of social connections, with one stating, “Social capital is, I would rank that as number one” (PLS 14), and another saying, “I would say social, in my opinion” (PLS 6). However, others maintained the hierarchy with social capital as second: “Humans is first, then social second, and economics is third” (PLS 3).

Respondents emphasized that these programs helped reduce isolation, facilitated mutual aid, and supported the emotional well-being of refugees and immigrants. One librarian noted the importance of social capital for fostering inclusion and safety: “Once they feel connected, they start to share resources and opportunities with each other. That can only happen in a space where they feel safe” (PLS 6). The physical design of libraries as open, non-stigmatized public spaces was also noted as conducive to social capital formation.

#### 4.3.3. Economic Capital: Toward Self-Sufficiency Through Library Services

Economic capital was typically ranked third but was still seen as essential for long-term stability. Participants discussed a range of library services that support financial independence, including job search assistance, resume writing help, access to job boards, and financial literacy classes. Interviewees confirmed the ranking of economic capital: “Economics is third” (PLS 3); “Economics is third” (PLS 1); and “Economics might be the third” (PLS 11).

However, many interviewees noted that economic gains are often delayed or limited without sufficient human and social capital. One participant explained, “Building on that with economics requires more translation services and things that we just don’t quite have the infrastructure for yet” (PLS 18). Another acknowledged the essential role of economic capital within livelihoods, stating, “Economics is, of course, part of people’s livelihood. So that is there too” (PLS 14).

Although economic capital was ranked lower, its importance was recognized in stories of transformation. Some librarians described how patrons who began with ESL or literacy classes later found employment and returned to the library to mentor others, illustrating how human and social capital serve as steppingstones to economic self-sufficiency.

#### 4.3.4. Refugee Success Story: Navigating Challenges and Achieving Integration

Across Oklahoma’s three library systems, refugees’ journeys toward successful integration reveal resilience, community support, and transformative experiences. A library staff member shared, “I was talking to a customer with her kids and a teenage boy who had recently relocated from Afghanistan. She shared that he’s improving his English and has developed a strong interest in graphic novels.” (PLS 15). The story highlights the family’s resilience in overcoming displacement challenges. The staff member further noted that “he’s improving his English and has developed a strong interest in graphic novels,” demonstrating the youth’s determination to engage with new languages and cultural forms.

Community support through libraries is central to this narrative. “He wants to read graphic novels. I took her over there to point her towards those graphic novels,” the librarian explained. Libraries serve as cultural hubs where refugees connect with services and educational opportunities, fostering inclusion and belonging.

While celebrating achievements, the story prompts reflection on long-term challenges. Another staff member (PLS 18) observed, “Sustained language support beyond initial stages and addressing socio-economic barriers are crucial,” highlighting the need for comprehensive support frameworks beyond immediate settlement.

### 4.4. RQ 4—What Comprehensive Guidelines and Strategies Can Be Implemented Within Public Libraries to Create Sustainable Pathways for Refugee Resilience?

Based on the interview data, enhancing library support for refugee and immigrant communities revolves around nine primary themes: (1) Community Engagement and Outreach; (2) Staff Engagement and Training; (3) Multilingual Resources and Services; (4) Advocacy and Funding; (5) Partnerships and Collaborations; (6) Program and Service Development; (7) Transportation; (8) Cultural Competence; and (9) Facility and Space Optimization (Table 3).

#### 4.4.1. Theme 1: Proactive Community Engagement and Outreach

Proactive community engagement is essential for libraries to effectively support refugee and immigrant populations. This involves deeply connecting with local communities to understand their unique needs and build meaningful relationships. As one participant highlighted, organizing “roundtables for working with an international population” is a crucial first step to identify and address these needs. Libraries should not wait for refugees to come to them but instead proactively reach out by “going out to the places where they already go and providing information there.” This outreach strategy ensures that library services are accessible and visible to the community. Sustained advocacy is also vital to secure funding and resources. Engaging elected officials and community stakeholders helps emphasize the importance of supporting refugees and immigrants. Building trust and rapport with community members, which often requires time, was noted as key: “it may just take for me personally, a little bit of time… to develop a rapport with them where I could say, hey, do you guys want a specific program that we could offer?” This personalized approach helps tailor services to meet community needs effectively.

#### 4.4.2. Theme 2: Staff Engagement and Enhancing Capacity for Inclusive Service Delivery

Staff training emerged as a key strategy for inclusive library design. When asked how to overcome challenges in making refugees feel welcome and informed, 78% of respondents pointed to a lack of knowledge about refugees’ needs. Nearly 80% stressed the importance of training staff in cultural awareness and multilingual communication to better support and engage refugee communities. As one participant shared, “We do not know what are we doing and whom we are doing” (PLS11), illustrating uncertainty around current practices. Participants emphasized that staff must be open to understanding the unique needs of refugee families. Programming should be relevant, targeted, and paired with individualized support. One-on-one assistance was frequently cited as an effective method for building trust and offering personalized services.

Training on engagement with non-native English speakers was repeatedly highlighted. One participant remarked, “Librarian staff needs training” (PLS 11), underlining the necessity of continuous professional development. Additionally, strategic hiring of bilingual staff was seen as vital. One interviewee noted her aim to “hire someone who is bilingual and speaks Zomi and English” (PLS 18), underscoring the importance of linguistic representation and tailored communication. Together, these findings suggest that investing in staff training and multilingual capacity is essential for public libraries to deliver responsive, equitable services to refugee families.

#### 4.4.3. Theme 3: Multilingual Resources and Services

Inclusive programming plays a pivotal role in fostering cultural understanding and integration. Bilingual storytimes and multicultural events were widely cited as effective strategies. One participant suggested, “bilingual storytime…to provide a space to learn and practice language skills,” emphasizing the dual benefits of language acquisition and cultural inclusion.

Opportunities for broader cultural sharing were also noted. An interviewee stated, “anytime you can sit down with somebody different from you and share your stories and culture, it can be very powerful in breaking down barriers.” These initiatives foster community cohesion and empower refugees to share their identities. Tailoring programs to address daily needs further enhances engagement; as one participant shared, they “provide programming geared towards communities within our catchment area,” highlighting the importance of localized service design.

#### 4.4.4. Theme 4: Advocacy and Funding

Advocacy and cross-sector collaboration emerged as vital strategies for strengthening refugee-focused library services. Participants emphasized partnering with nonprofit organizations and community leaders to introduce refugees to the library. As one participant noted, “nonprofits helping refugees…introduce them to the library,” pointing to the role of partnerships in creating access pathways. Securing funding and integrating staff training were also prioritized. One interviewee stressed, “We must have knowledgeable and trained staff on how to address those needs as quickly as possible” (PLS 8), highlighting the need for proactive capacity building.

Leadership and advocacy were repeatedly emphasized as central to sustainability. A participant stated, “you need an advocate to lead the work and be responsible for ushering in the growth needed to sustain programs that support new communities” (PLS 11). Another underscored, “advocating for advocacy is a huge solution, talking with your elected officials” (PLS 8), illustrating libraries’ role in influencing resource allocation and public support.

#### 4.4.5. Theme 5: Partnerships and Collaboration

Effective collaboration with nonprofit organizations and community leaders is critical in introducing refugees to library services. One interviewee suggested, “nonprofit organizations that are helping the refugees…if those nonprofits could introduce them to the library, perhaps do some of their meetings in the library to just make them familiar with the location, then we can give them an introduction to what the library has for them as far as resources are concerned.” Engaging with community leaders who are already trusted within refugee communities can amplify the library’s outreach efforts. An interviewee noted, “connecting with community leaders that exist in these communities already, making sure that they’re aware of all the things that we’re doing and all the things we want to do for these communities.”

#### 4.4.6. Theme 6: Program and Service Development

Libraries are increasingly prioritizing job training and skill development programs tailored to the specific needs of refugee and immigrant communities. As one interviewee shared, “we try to develop skills… helping develop skills or knowledge on how to behave or when they go to get a real job” (PLS 13). This reflects a shift toward practical support services that facilitate workforce integration. A proactive approach to community engagement was highlighted by another participant who stated, “our goal… is to provide a resource to meet the community needs” (PLS 13). Libraries are not only responding to expressed needs but also anticipating future demands to better serve their patrons. Positioned as agents of upward mobility, libraries are seen as playing a critical role in economic empowerment. As one interviewee expressed, “we want to do more for our community and serve upward mobility,” signaling a broader institutional commitment to long-term support and development (PLS 5). In alignment with this vision, specific initiatives such as “providing job training skills, resume writing, and cover letter writing skills” were emphasized as essential tools for securing employment and fostering career advancement (PLS 15).

#### 4.4.7. Theme 7: Transportation Solution

To address transportation challenges, libraries can implement several strategies informed by interview insights. Partnering with local transportation authorities could improve accessibility; as one participant noted, confusing bus routes often create barriers to library access. Libraries might advocate for more intuitive routes or offer transportation subsidies to better connect refugee communities to library services. Mobile library services also emerged as a promising solution, bringing resources directly into refugee neighborhoods. This reflects an outreach-focused philosophy, as one librarian explained: “Going out to the places where they already go and providing that information to them there” (PLS 6). Expanding digital resources and virtual programming offers another way to mitigate transportation barriers by enabling access without requiring physical presence. Additionally, libraries could consider strategic planning of new branches or satellite locations in areas with high refugee populations and limited public transit access (Table 3).

#### 4.4.8. Theme 8: Cultural Competence and Fostering Awareness

Raising awareness about library services through various communication channels is essential. Libraries can use social media, multilingual posters, and community events to inform refugees about available resources. One interviewee suggested, “Offering just like social media or posters around the library can be done.” Effective communication strategies help create an inviting and supportive environment for refugees. Prioritizing the hiring of bilingual staff and providing resources in multiple languages can significantly enhance the accessibility of library services. Comprehensive training for library staff on cultural competency and communication with non-English speakers is crucial.

#### 4.4.9. Theme 9: Fostering Welcoming Environments

Creating a welcoming environment is essential for ensuring accessibility and inclusivity within libraries. This includes prioritizing the hiring of bilingual staff and providing resources in multiple languages. A participant suggested using “posters throughout the library…in other languages to draw people in.” Effective communication in diverse languages helps to bridge communication gaps and make library services more accessible to refugee communities.

Offering educational programs tailored to refugee children supports their integration and development. “Offering opportunities for kids to be kids here is something that we do,” one interviewee emphasized, highlighting the importance of providing safe and supportive spaces within libraries.

**Figure 4:**
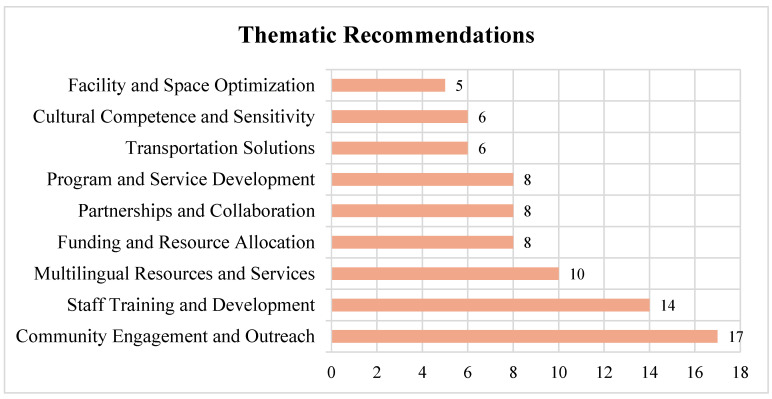
Libraries’ facilities improvement recommendations.

Overall, we found that community engagement and outreach (17 mentions) and staff training and development (14 mentions) were the most frequently recommended areas for improvement, highlighting the libraries’ emphasis on building trust and cultural competence when serving refugee communities (Figure 4). Multilingual resources and services, along with program and service development, partnerships and collaboration, and funding and resource allocation were also notable priorities, reflecting the need for inclusive services and sustainable support structures. While facility and space optimization, cultural competence and sensitivity, and transportation solutions received fewer mentions, they remain important considerations for im-proving access and comfort. These findings underscore a multifaceted approach to making libraries more inclusive, equitable, and trauma-informed for refugee populations.

## 5. Theorizing and Interpretation

This study underscores the transformative yet often underrecognized role public libraries play in refugee resettlement, positioning them as adaptive, trauma-informed infrastructures that mediate not just access to services, but also the broader processes of healing, identity reconstruction, and social belonging. Drawing from the lived insights of library professionals in Oklahoma, the findings offer a multi-scalar view of how libraries help rebuild the fractured ecosystems of refugees’ lives.

In response to the first research question—what services and programs public libraries provide—the findings revealed cognitive services—including language acquisition, digital literacy, and access to multilingual resources—play a foundational role in developing human capital. These offerings mirror findings from prior studies [6,21] and reflect libraries’ capacity to facilitate both individual learning and family-level empowerment. Importantly, such services not only support educational attainment but also serve as entry points into broader systems of economic and social participation.

Beyond cognition, socio-cultural services position libraries as relational institutions that nurture belonging, place attachment, and trust. The data confirms that libraries are perceived as democratic, low-barrier spaces where refugees form new connections, share culture, and gradually integrate into the fabric of their host communities. This aligns with previous research [2,85,86], which positions libraries as informal arenas for cultural exchange, civic participation, and identity reconstruction. However, the political dimensions of integration—such as civic engagement and advocacy—remain underdeveloped in many library systems, suggesting an area for growth.

The physiological dimension, though often overlooked in library studies, was especially salient in this research. Libraries offer physical and emotional refuge—safe, climate-controlled environments where families gather, children play, and individuals rest. Design elements, from calming interiors to storytelling spaces, reinforce feelings of comfort and safety. This suggests libraries are not just sites of service, but therapeutic environments contributing to the psychosocial well-being of resettled populations. These findings extend earlier frameworks [87,88] by highlighting how material and spatial design influences user experience, especially among trauma-affected groups.

The second research question focused on how public libraries influence refugee resilience and integration. Participants identified libraries as spaces where refugees could rebuild human capital—such as language acquisition, digital literacy, and formal education—while also generating social capital through relationships, trust-building, and engagement with both co-ethnic and host communities. Libraries also supported economic capital by offering job readiness training, resume workshops, and computer access. These overlapping forms of capital are critical to fostering long-term resilience. Rather than treating integration as a linear process, the findings support a multidimensional view in which libraries actively cultivate resources that enable refugees to adapt, participate, and thrive. In this light, libraries function not merely as service providers but as social infrastructures that facilitate agency, dignity, and psychosocial recovery.

Despite these strengths, the third research question surfaced persistent challenges that constrain libraries’ ability to fully support refugee communities. These include limited budgets, insufficient staffing—especially in terms of bilingual or culturally responsive personnel—language and cultural barriers, and a lack of strategic outreach or political engagement mechanisms that would allow refugee voices to be more actively included in decision-making also align with previous study [12]. Collaboration with local, national, or international entities is crucial in designing inclusive public libraries, considering displaced people’s location to encourage their involvement. Maximizing human and financial resources and creating stronger ties with displaced people should be prioritized [88]. Such constraints reflect broader systemic inequities that are often embedded within public institutions and raise concerns about sustainability, equity, and accessibility. Without addressing these structural limitations, the transformative potential of libraries risks remaining uneven or unrealized.

The fourth research question asked what strategies and recommendations could enhance libraries’ role in refugee support. Interview participants articulated that culturally competent hiring, trauma-informed staff training, and embedded partnerships with resettlement agencies represent more than administrative improvements; they reflect a commitment to building libraries as participatory, equitable, and anti-oppressive infrastructures. These strategies highlight a paradigm shift from viewing refugee patrons as service users to recognizing them as knowledge holders, collaborators, and contributors to civic life.

The success stories documented in this study demonstrate how libraries can positively impact individual refugee experiences. From Afghan teenagers discovering graphic novels to families accessing crucial citizenship resources, these narratives illustrate the transformative potential of library services when delivered through culturally responsive approaches. As Oklahoma continues to welcome diverse refugee populations, public libraries stand positioned to play an increasingly significant role in fostering resilience and supporting successful integration. By implementing the comprehensive guidelines and strategies identified in this study, libraries can create sustainable pathways for refugee resilience that extend beyond immediate settlement needs to long-term community belonging and socioeconomic participation. The development of inclusive library services represents not only an investment in refugee communities but also an enrichment of the broader social fabric of Oklahoma.

In synthesizing across the four research questions, broader insight surfaces: libraries are not just sites of integration, but sites of resilience reconstruction. They mediate critical dimensions of ecological recovery—cognitive, emotional, social, and physical, particularly when institutional actors are empowered with the resources, frameworks, and partnerships to do so.

Critically, the study affirms that resilience is not an individual trait, but an ecological process built through community, access, and care. Libraries foster this resilience not through one-time interventions, but through sustained, equitable relationships with refugee communities. They do so by recognizing refugees as agents of knowledge and contributors to the civic fabric—not just service recipients. However, libraries operate within broader institutional and funding constraints that affect their capacity to implement inclusive library design and service models [89].

## 6. Strengths, Weaknesses, and Future Research Directions

This study offers an in-depth, context-specific understanding of how public libraries in Oklahoma support refugee resilience through a holistic approach encompassing cognitive, socio-cultural, and physiological dimensions. The use of semi-structured interviews with library staff across multiple systems provides rich, practitioner-informed insights often missing in the literature. The research highlights the multifaceted role libraries play beyond traditional service delivery, emphasizing their potential as trauma-informed and community-centered spaces. The study is limited by its qualitative design and regional focus, which may affect the generalizability of findings to other geographic or institutional contexts. Additionally, while library staff perspectives are central, the voices of refugee patrons themselves were not included, potentially limiting the understanding of user experiences. Systemic factors such as governance and funding structures were only indirectly addressed, leaving gaps in assessing institutional constraints.

Future studies should incorporate refugee users’ perspectives to deepen understanding of lived experiences and needs. Quantitative research could measure the impact of library programs on refugee integration outcomes more systematically. Investigating the role of institutional policies, funding mechanisms, and cross-sector partnerships would illuminate structural enablers and barriers to inclusive library service.

## 7. Conclusions

Oklahoma’s public libraries are quietly but powerfully shaping the refugee experience, serving as multi-dimensional support systems that foster resilience, belonging, and long-term integration. Through cognitive offerings such as language and digital literacy classes, socio-cultural programming that promotes connection and trust, and physiological environments that prioritize safety and comfort, libraries are reimagining what inclusive, trauma-informed public service looks like.

Yet, this transformative work occurs despite systemic challenges—understaffing, limited funding, and barriers to refugee engagement. To fulfill their potential as civic anchors, libraries must be more explicitly resourced and recognized as key players in refugee resettlement ecosystems. This includes not only expanding services but embedding principles of equity, inclusion, and co-creation into their institutional frameworks. As refugee populations continue to grow and diversify, public libraries offer a replicable model for socially just, locally grounded integration. Their strength lies not in grand gestures, but in everyday acts of access, empathy, and community-building. Supporting libraries is thus not only a commitment to displaced individuals, but also an investment in the collective well-being and democratic future of our communities.

## Figures and Tables

**Table 1:** Participants’ Profile.

Interviewee ID	Interviewee’s Designation	Interviewee’s Contact	Library System	Work Experiences	Gender
PLS 1	Early Childhood Librarian	Downtown Library	MLS	5–10 yrs.	(she/her)
PLS 2	Information Services Department Manager	Pioneer Lib-Central	PLS	5–10 yrs.	(he/him)
PLS 3	Selector	Pioneer Lib-West	PLS	5–10 yrs.	(she/her)
PLS 4	Department Manager	Pioneer Lib–Central	PLS	5–10 yrs.	(she/her)
PLS 5	Library Associate	Moore Public Library	PLS	5–10 yrs.	(she/her)
PLS 6	Library Associate	Moore Public Library	PLS	5–10 yrs.	(he/him)
PLS 7	Outreach Specialist	Moore Public Library	PLS	5–10 yrs.	(she/her)
PLS 8	Director of Communications & Employee Development	Pioneer Lib-Central	PLS	5–10 yrs.	(she/her)
PLS 9	Assistant Library Manager	Ralph Ellison Library	MLS	5–10 yrs.	(she/her)
PLS 10	Children’s Librarian I	Southern Oaks Library	MLS	5–10 yrs.	(she/her)
PLS 11	Programs Manager Outreach & Engagement Services	Metropolitan Library	MLS	5–10 yrs.	(she/her)
PLS 12	Library Associate	Belle Isle Library	MLS	5–10 yrs.	(she/her)
PLS 13	Ex-library Staff	Village Library	MLS	2–5 yrs.	(she/her)
PLS 14	Branch Manager	Pioneer Public Lib-East	PLS	5–10 yrs.	(she/her)
PLS 15	Manager-Central Info. Services	Metropolitan Library	MLS	5–10 yrs.	(he/him)
PLS 16	Engagement Specialist II	Capitol Hill Library	MLS	1–2 yrs.	(she/her)
PLS 17	Literacy Outreach & Volunteer Services	Tulsa City- County Library	TCCL	5–10 yrs.	(she/her)
PLS 18	Manager	Jenks Library	TCCL	5–10 yrs.	(she/her)
PLS 19	Assistant Manager—Children’s Department	Tulsa City- County Library	TCCL	5–10 yrs.	(she/her)
PLS 20	Branch Manager	Glenpool Library	TCCL	5–10 yrs.	(he/him)

(Source: Authors, 2023).

**Table 2:** Challenges in providing services to refugee communities.

Interviewee ID	Interviewee’s Contact	Key Challenges
PLS 1	Downtown Library	Limited availability of multilingual materials like Pashto and Dari Materials. Difficulty in Obtaining MARC Records (Consider outsourcing cataloging for non-bilingual materials)
	Pioneer Lib-Central	Awareness; Communication Barriers; Cultural Sensitivity
PLS 3	Pioneer Lib-West	Awareness and Outreach; Resource Constraints (staff and technology)
PLS 4	Pioneer Lib-Central	Reaching out to refugee families
PLS 5	Moore Public Library	Cultural and Contextual Understanding (Lack of Direct Experience; Staff Expertise); Community Engagement and Trust
PLS 6	Moore Public Library	Transportation; Facility Limitations; Program and Service Gaps; limited computer and Digital Access; No Tutoring Services,
PLS 7	Moore Public Library	Difficulty in communication between library staff and refugee children due to the lack of multilingual staff or language proficiency; Transportation; The library’s old building lacks sufficient space and modern amenities to accommodate the needs of all users, Outreach; Staff Training; Economic Development Programs
PLS 8	Pioneer Lib-Central	Funding Constraints; Transportation Challenges; Library Placement; Internet Connectivity
PLS 9	Ralph Ellison Library	Connect with Refugee families; transportation and Location; Creating programs specifically for refugee children without excluding other community members can be difficult, Understanding Needs; Lack of Data; Privacy Concerns; Identifying Refugee Families
PLS 10	Southern Oaks Library	Lack of Awareness; Refugee families often do not know what services are available at the public library; Language Barriers; Insufficient translation of written communications and materials can hinder access; Transportation and Location; Program Accessibility; Program Accessibility (Insufficient Data)
PLS 11	Metropolitan Library	Funding Challenges: Education and Training
PLS 12	Belle Isle Library	Utilization of Library Spaces, Location and Transportation; Economic Growth Measurement.
PLS 13	Village Library	Identifying Refugees; Lack of Specialized Programs; Collaboration with Organizations; Insufficient Partnerships
PLS 14	Pioneer Public Lib-East	Identification and Outreach (Identifying Refugees; Awareness and Reach); Program Development and Resources; Language and Cultural Barriers (Language Accessibility; Cultural Competence)
PLS 15	Metropolitan Library	Identification and Outreach Challenges; Limited Outreach; Language and Cultural Barriers; Program Development and Resources; Resource Constraints
PLS 16	Capitol Hill Library	Lack of Staff Diversity; Misunderstanding of Library Services; socially unsafe or unwelcome; Limited Public Transportation; Geographic Disparities;
PLS 17	Tulsa City-County Library	Financial Constraints; Limited Funding; Staff Availability; Understanding Community Needs;
PLS 18	Jenks Library	Human Capital and Staffing Constraints; Transportation Barriers; Limited Staff Resources; Financial Limitations
PLS 19	Tulsa City-County Central Library	Community Awareness and Engagement; Identifying Refugee Populations; Cultural and Linguistic Barriers; Budget Limitations; Staffing and Training; Physical Accessibility; transportation barriers
PLS 20	Glenpool Library	Visibility and Outreach; Transportation Access; Walking Distance Considerations

**Table 3:** Recommendations and Interviewees’ Profile.

Interviewee ID	Interviewee’s Designation	Interviewee’s Contact	Key Recommendations
PLS 1	Early Childhood Librarian	Downtown Library	Networking; ALA and Vendor Engagement; Diversity in Collections
PLS 2	Information Services Department Manager	Pioneer Lib-Central	Community Engagement; Multilingual Resources; Tech Integration; Tailored Programs
PLS 3	Selector	Pioneer Lib-West	Outreach; Mobile Library Services; Community Partnerships
PLS 4	Department Manager	Pioneer Lib-Central	Community Partnerships; Education Programs; Targeted Outreach
PLS 5	Library Associate	Moore Public Library	Diversity Training; Staff Hiring; Cultural Events; Community Collaboration
PLS 6	Library Associate	Moore Public Library	Transportation Access; Mobile Services; Tutoring; NGO Collaboration
PLS 7	Outreach Specialist	Moore Public Library	Multilingual Staff; Transit Passes; Virtual Programs; Career Workshops
PLS 8	Director of Communications & Employee Development	Pioneer Lib-Central	Fundraising; 24-Hour Access; Digital Expansion; Listening to Community
PLS 9	Assistant Library Manager	Ralph Ellison Library	Privacy; Inclusive Programming; Location Planning; Transit Support
PLS 10	Children’s Librarian I	Southern Oaks Library	Multilingual Outreach; Transportation; Feedback; Community Awareness
PLS 11	Programs Manager Outreach & Engagement Services	Metropolitan Library	Grant Access; Sustainable Funding; Ongoing Staff Training
PLS 12	Library Associate	Belle Isle Library	Facility Optimization; Transportation; Collaborative Outreach
PLS 13	Ex library Staff	Village Library	Program Identification; Partnerships; Grant and Philanthropy Support
PLS 14	Branch Manager	Pioneer Public Lib-East	Cultural Accessibility; Tailored Programs; Evaluation; Staff Training
PLS 15	Manager-Central Information Services	Metropolitan Library	Targeted Outreach; Translation Services; Evaluation; Staff Support
PLS 16	Engagement Specialist II	Capitol Hill Library	Staff Diversity; Multilingual Materials; Resettlement Partnerships
PLS 17	Literacy Outreach & Volunteer Services	Tulsa City- County Library	Tailored Services; Staff Development; Funding Strategies; Feedback
PLS 18	Manager	Jenks Library	Advocacy; Responsive Programs; Staff Training; Community Outreach
PLS 19	Assistant Manager—Children’s Department	Tulsa City- County Central Library	Language Services; Culturally Sensitive Programs; Awareness
PLS 20	Branch Manager	Glenpool Library	Mobile Outreach; Youth Focus; Safety; Transportation Access

## Data Availability

The Data is not available for privacy or ethical restrictions.
